# Supplementary material for: Mechanisms governing the pioneering and redistribution capabilities of the non-classical pioneer PU.1
Source: Nat Commun. 2020 Jan 21;11:402. doi: 10.1038/s41467-019-13960-2 (PMC6972792; doi:10.1038/s41467-019-13960-2)
Supplement: Supplementary file 7 — Source data [file 41467_2019_13960_MOESM7_ESM.zip › Source_Data/FigureS2/FigureS2B_MotifScanOutput/KmeansCluster1/homerResults/motif11.similar.html]

motif11

## Information for motif11

A
G
T
C
A
C
G
T
A
C
G
T
A
G
T
C
A
G
T
C
A
G
C
T
A
C
G
T
  
Reverse Opposite:  

C
G
T
A
C
T
G
A
A
C
T
G
A
C
T
G
C
G
T
A
C
G
T
A
A
C
T
G
  

|  |  |
| --- | --- |
| p-value: | 1e-89 |
| log p-value: | -2.065e+02 |
| Information Content per bp: | 1.945 |
| Number of Target Sequences with motif | 1165.0 |
| Percentage of Target Sequences with motif | 22.88% |
| Number of Background Sequences with motif | 4754.0 |
| Percentage of Background Sequences with motif | 11.99% |
| Average Position of motif in Targets | 97.5 +/- 51.5bp |
| Average Position of motif in Background | 105.0 +/- 99.1bp |
| Strand Bias (log2 ratio + to - strand density) | 0.3 |
| Multiplicity (# of sites on avg that occur together) | 2.13 |
| Motif File: | file (matrix) reverse opposite |

### Similar de novo motifs found

|  |  |  |  |  |  |  |  |
| --- | --- | --- | --- | --- | --- | --- | --- |
| Rank | Match Score | Redundant Motif | P-value | log P-value | % of Targets | % of Background | Motif file |
| 1 | 0.905 | C G T A A C T G A C T G A C T G C G T A C G T A A C T G | 1e-86 | -198.695949 | 19.66% | 9.75% | motif file (matrix) |
| 2 | 0.757 | C A T G C T G A T C G A T C A G T C A G C T G A T C A G T C A G | 1e-42 | -98.344848 | 7.62% | 3.27% | motif file (matrix) |
| 3 | 0.885 | C G T A C T A G A T C G A C T G C T G A C T G A A C T G C T A G | 1e-14 | -33.741642 | 15.18% | 11.29% | motif file (matrix) |
